# Frontiers of Robotic Colonoscopy: A Comprehensive Review of Robotic Colonoscopes and Technologies

**DOI:** 10.3390/jcm9061648

**Published:** 2020-05-31

**Authors:** Gastone Ciuti, Karolina Skonieczna-Żydecka, Wojciech Marlicz, Veronica Iacovacci, Hongbin Liu, Danail Stoyanov, Alberto Arezzo, Marcello Chiurazzi, Ervin Toth, Henrik Thorlacius, Paolo Dario, Anastasios Koulaouzidis

**Affiliations:** 1 The BioRobotics Institute, Scuola Superiore Sant’Anna, 56025 Pisa, Italy; veronica.iacovacci@santannapisa.it (V.I.); marcello.chiurazzi@santannapisa.it (M.C.); paolo.dario@santannapisa.it (P.D.); 2 Department of Excellence in Robotics & AI, Scuola Superiore Sant’Anna, 56127 Pisa, Italy; 3 Department of Human Nutrition and Metabolomics, Pomeranian Medical University in Szczecin, 71-460 Szczecin, Poland; karzyd@pum.edu.pl; 4 Department of Gastroenterology, Pomeranian Medical University in Szczecin, 71-252 Szczecin, Poland; marlicz@hotmail.com; 5 Endoklinika sp. z o.o., 70-535 Szczecin, Poland; 6 School of Biomedical Engineering & Imaging Sciences, Faculty of Life Sciences and Medicine, King’s College London, London SE1 7EH, UK; hongbin.liu@kcl.ac.uk; 7 Wellcome/EPSRC Centre for Interventional and Surgical Sciences (WEISS), University College London, London W1W 7TY, UK; danail.stoyanov@ucl.ac.uk; 8 Department of Surgical Sciences, University of Torino, 10126 Torino, Italy; alberto.arezzo@unito.it; 9 Department of Gastroenterology, Skåne University Hospital, Lund University, 20502 Malmö, Sweden; ervin.toth@med.lu.se; 10 Department of Clinical Sciences, Section of Surgery, Lund University, 20502 Malmö, Sweden; henrik.thorlacius@med.lu.se; 11 Endoscopy Unit, The Royal Infirmary of Edinburgh, Edinburgh EH16 4SA, UK; akoulaouzidis@hotmail.com

**Keywords:** colorectal cancer screening, robotic colonoscopy, artificial intelligence, computer-aided diagnosis for colonoscopy, robotics for medical distancing

## Abstract

Flexible colonoscopy remains the prime mean of screening for colorectal cancer (CRC) and the gold standard of all population-based screening pathways around the world. Almost 60% of CRC deaths could be prevented with screening. However, colonoscopy attendance rates are affected by discomfort, fear of pain and embarrassment or loss of control during the procedure. Moreover, the emergence and global thread of new communicable diseases might seriously affect the functioning of contemporary centres performing gastrointestinal endoscopy. Innovative solutions are needed: artificial intelligence (AI) and physical robotics will drastically contribute for the future of the healthcare services. The translation of robotic technologies from traditional surgery to minimally invasive endoscopic interventions is an emerging field, mainly challenged by the tough requirements for miniaturization. Pioneering approaches for robotic colonoscopy have been reported in the nineties, with the appearance of inchworm-like devices. Since then, robotic colonoscopes with assistive functionalities have become commercially available. Research prototypes promise enhanced accessibility and flexibility for future therapeutic interventions, even via autonomous or robotic-assisted agents, such as robotic capsules. Furthermore, the pairing of such endoscopic systems with AI-enabled image analysis and recognition methods promises enhanced diagnostic yield. By assembling a multidisciplinary team of engineers and endoscopists, the paper aims to provide a contemporary and highly-pictorial critical review for robotic colonoscopes, hence providing clinicians and researchers with a glimpse of the major changes and challenges that lie ahead.

## 1. Introduction

### 1.1. Medical Needs and Clinical Aspects in Colonoscopy

**Figure 1:**
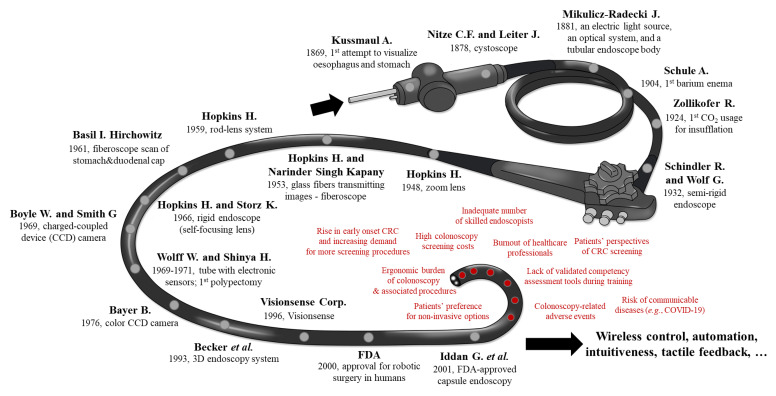
Graphical representation of all the key milestones in colonoscopy (black text) and possible factors impeding future high-throughput colonoscopy screening programs (red text).

Colon, lung, and female breast cancers are responsible for one third of cancer-related deaths worldwide. According to the GLOBOCAN 2018 report, the number of estimates of colorectal cancer (CRC) incidence and mortality reported by the International Agency for Research on Cancer (IARC), drastically increased. With almost 900,000 deaths annually, CRC is the third most diagnosed neoplasm and the second leading cause of cancer-related death worldwide [1], with the highest incidence in Europe, Australia, New Zealand, North America and Asia. The incidence of CRC mirrors economic development, westernized dietary and lifestyle changes and can be viewed as a marker of socioeconomic development [2]. The rising prevalence of obesity is an important risk factor for the early onset of CRC with increasing occurrence in individuals younger than 50 years. While lifestyle changes and new cancer management strategies led to a decrease in CRC incidence and mortality in the United States, France and Japan, in other countries these are still on the rise [1]. Early-stage CRC detection, mainly through colonoscopy screening programs, contributed significantly to a higher survival rate [3] with an improved quality of life [4]. However, screening programs have already been or will be impeded in the near future by psychosocial, demographic and other healthcare related factors, including: (1) high colonoscopy screening costs, especially in low and middle-income countries or nations where other frequent diseases challenge healthcare budgets, (2) risk of communicable disease transmission, e.g., viral infections such as coronavirus SARS-CoV-2 (severe acute respiratory syndrome coronavirus-2), (3) rising incidence of early onset CRC and environmental risk factors, (4) inadequate number of skilled healthcare professionals, (5) long learning curves to achieve full professional experience in endoscopic techniques, (6) increasing numbers of physicians/nurses with burnout syndromes, (7) ergonomic burdens of colonoscopy and associated procedures, (8) increasing number of “need-to-screen” individuals worldwide, (9) lack of validated competency assessment training tools, and (10) patients’ perspective on CRC screening with preferences to choose non-invasive options [5,6,7,8,9,10,11] (see Figure 1, red text).

The issue of adverse events associated with an invasive procedure, such as colonoscopy, is another important limiting point. While, the risk of colonoscopy related perforation is low, it remains stable over time and independent of the endoscopists’ experience [12,13]. Lin et al., in a large cohort of patients (*n* = 112,543 who underwent colonoscopy or sigmoidoscopy and 112,543 matched patients not undergoing these procedures) reported the increased risk of infection (9.38-fold risk of infection; 95% confidence interval, 6.81–12.93; *p* < 0.001, including diverticulitis, peritonitis and appendicitis) in comparison to a control group [14]. Of all the above mentioned impeding factors, authors will briefly focus in this paper on the ergonomic burdens of endoscopists-performing standard colonoscopies and further discuss the opportunities and challenges to overcome obstacles in order to formulate efficacious, new generation robotic CRC high-throughput screening programs based on the robotic colonoscopy (RC) of the future.

### 1.2. Ergonomics: The Problem of Musculoskeletal Injuries

Colonoscopy forms a significant portion of endoscopists’ workload. However, not enough attention is given to the ergonomic aspects of conventional colonoscopy. Common anatomical sites of work-related musculoskeletal pains are the back (15–57%), neck (9–46%), shoulders (9–19%), elbows (8–15%) and hands/fingers (14–82%) [15]. However, this burden is not only limited to practicing gastrointestinal (GI) endoscopy; it seems to be also a common place among gastroenterology/endoscopy trainees or fellows [16,17].

Promoting a culture of fitness-conscious and regular-exercising professionals could reduce or delay the impact of high-volume colonoscopy workload on muscle overuse. Gender and anthropomorphic features should also be considered together with basic recommendations on the monitors positioning and the examination’s bed height in order to minimize endoscopy-related musculoskeletal injuries. Furthermore, limited data show that injuries of the hand, wrist, forearm and shoulder are most common among colonoscopists and may derive from general overuse of the upper limbs, repetitive pinching, gripping and torqueing forces and/or awkward neck and body posturing [18]. Pinch forces and forearm-muscle loads applied during routine colonoscopies represent substantial risk factors for carpal tunnel syndrome, de Quervain syndrome and/or tennis elbow.

Although the introduction of advanced colonoscopes and insertion techniques, such as water-assisted or gasless colonoscopy, could help not only with patient comfort and/or increased polyp detection but also with effort reduction on the side of the endoscopist, their adoption is far from being “universal”. Therefore, a paradigm shift is required in devices and techniques to improve safety and comfort and to ensure uninterrupted, efficient and high-quality provision of endoscopy services in the face of rising demand worldwide for both screening and therapeutic colonoscopy [15].

### 1.3. History and Key-Milestones of Colonoscopy

It took almost one century before the first endoscopic attempts to visualise GI tract were performed and a few decades since 1904 when the first barium enema examination was performed [19,20] to a full retrograde flexible colonoscopy [21]. Soon after it, in 1969, endoscopic excision of colonic polyps was possible. Dr. Hiromi Shinya, a newly qualified general surgeon at Beth Israel Medical Center (New York, NY, USA) and Dr. William Wolff, Chairman of the General Surgery department, at that time were at the forefront of a worldwide research effort to develop ways to examine the full length of the colon using a tube embedding electronic sensors [22]. In 1969, the two pioneers made a further ground-breaking advancement in collaboration with Olympus Corp. (Tokyo, Japan) by introducing a wire loop snare to cauterize a polyp as soon as it was found, thus making a second procedure unnecessary [23]. Although the two advocated for their invention, they had to overcome some serious scepticism about the device’s safety and efficacy. Furthermore, the development of a protocol for a one-doctor technique, as the standard for performing colonoscopy, has been formally attributed to them.

The first flexible endoscopes included a fibre optic bundle. Approximately 250,000 glass fibres, each about 15μm in diameter, individually coated and oriented similarly at both ends, were placed in a 4 mm bundle that allowed the transmission of a visual image [24,25]. By 1973, Dr. Wolff and Dr. Shinya had performed over 2,000 colonoscopies in the Endoscopy Unit at Beth Israel Medical Center, demonstrating that, in skilled hands, this procedure could be done safely [26]. Since then, several steps allowed the adoption in the clinical practice of current state-of-the-art HD colonoscopes without magnification capabilities and with image enhancement modes. However, one factor that remains unaltered is that the single operator must undergo extensive practice and training to gain credentials for provision of comfortable and safe colonoscopy. Nevertheless, with the advent of miniaturization, wireless control and artificial intelligence (AI) -aided digestive tract “scope” will continue to develop. A schematic illustration of history and milestones of colonoscopy is reported in Figure 1.

### 1.4. What Is Robotic Colonoscopy and Why Is Now the Time?

In recent decades, the foremost general drive to develop robots was the need to drastically improve human safety in hazardous environments and/or to enhance human operator ability in medical procedures by reducing fatigue. Furthermore, there was an ever-growing desire to develop products with wider potential markets aimed at improving the quality of everyday life. A common denomination of such application scenarios was the need to operate in a scarcely structured environment, which ultimately requires increased abilities, a multimodality “constellation” of sensors and a higher degree of dexterity and autonomy [27].

Creating a parallelism with computer-assisted surgery, robots in colonoscopy are computer-integrated intelligent machines able to: (1) improve the safety and performances of standard healthcare provisions in diagnosis and therapy, such as precision, effectiveness, safety and reliability, (2) enhance interventional abilities of endoscopists and standardize their ability to operate, also in teleoperation, (3) reduce the daily workload with better ergonomics, and (4) augment the field of possible interventions [28]. Thanks to specific regulations and standards, i.e., the new Medical Device Regulation (European Union MDR 2017/745) and ancillary directives, today classification and methodological design guidelines and functioning tests are clearly identified for guaranteeing a high level of standardization, safety and efficiency of newly introduced medical robotic devices. In order to understand the role of a robot in medicine, and in particular in colonoscopy, we need to answer a few questions, such as:

- “What is the difference between non-robotic instrumental colonoscopes (Section 2) and robotic flexible colonoscopes (Section 3 and Section 4)?” Although there is not a single definition that will cover all aspects, we hope—at least—that this contribution will continue the conversation on this debated issue. For the authors, the difference between them is nestled in its intrinsic capability to enable and perform controlled assisted actions or autonomous procedures in an unstructured deformable environment, such as in the colonic tract.
- “What are the modules needed to achieve that?” Not only embedded sensors, such as the vision camera into the PillCam™ capsule or pH/temperature sensors are needed but also a complex hardware and software architecture that enables computer-integrated modalities, i.e., the information collected by sensors, through wired or wireless communications, can be elaborated thanks to AI algorithms (Section 5) for enabling advanced and potentially-autonomous actuation and actions (i.e., navigation of the device but also activation of mechanisms for drug-delivery and tissue sampling) in a closed-loop manner.
- “Why is now the time?” Technologies are now in a mature state and thanks to the wider use of robotic systems and technologies in surgery, endoscopists are now more open in accepting and collaborating with robotic companions during their activities [29,30]. In addition, under the current circumstances, one could in parallel to the term “social distancing” coin the term “medical distancing” (not in care, emotion or relationship but more in a physical contact during medical practices) via complex personal protective equipment or very simply reducing handshakes and consultation distance. Obviously, most of us believe that the end of the SARS-CoV-2 pandemic will allow things to go back to normal, however, this global pandemic sets the scene for innovation in ways and speed that have not been seen before in the field of minimally-invasive surgery and/or in remote robotic diagnosis and therapy in medicine. So, our last question is “Is it now the time for introducing in the medical practice a new teleoperated or even autonomous robotic colonoscope?”

The paper is organized as follows: Section 2 describes non-robotic colonoscopes and colonoscopy adjuncts used in the clinical practice, Section 3 summarizes the robotic flexible colonoscopes, as commercially-certified instruments, whereas Section 4 describes research-oriented innovative robotic colonoscopes. Moving to software development, Section 5 focuses on the potentialities of artificial intelligence tools in enhancing robotic colonoscopy and, finally, Section 6 reports discussions and conclusions of this comprehensive review paper.

## 2. Non-Robotic Colonoscopes and Colonoscopy Adjuncts in the Clinical Practice

Thus far, standard colonoscopy (SC) is considered the most effective methodology to diagnose CRC. Indeed, this method represents the gold standard practice for the evaluation of a wide range of colonic pathologies, due to its ability to visualize the internal surface of the colon, to acquire tissue samples and to treat precursors and early-stage tumours. However, (1) perceived invasiveness, (2) patient discomfort and/or fear of pain, hence, need for conscious sedation, and (3) the concern of social/medical distancing at a time of a pandemic, limit (or will limit for the latest) the use of screening colonoscopy [31]. The population does not participate in screening programs because colonoscopy itself and the necessary preparation of the intestine by dietary adjustment and numerous laxatives are perceived as painful and not worth it by many people [32,33].

**Figure 2:**
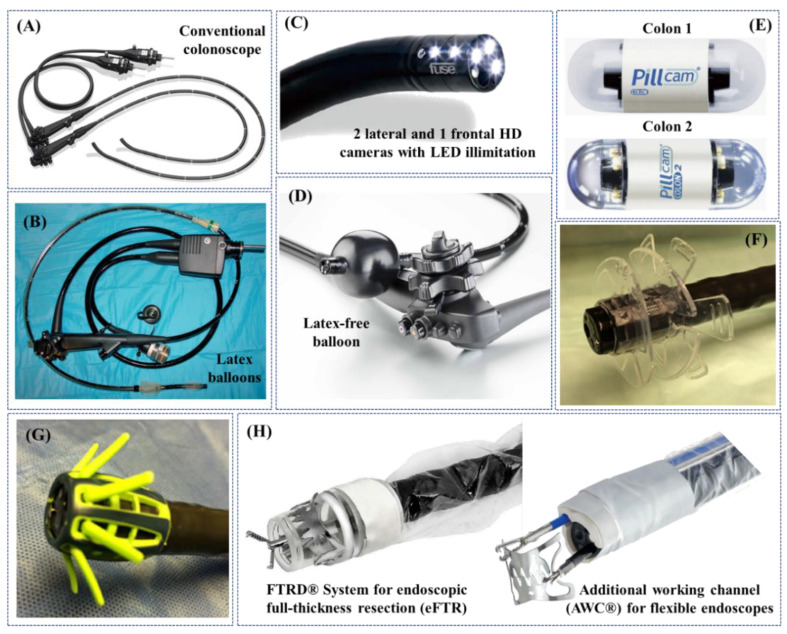
Examples of non-robotic colonoscopes and adjuncts used in the clinical practice: (**A**) standard colonoscope; (**B**) double-balloon colonoscope [47]; (**C**) Full Spectrum Endoscopy (FUSE, EndoChoice Inc., Alpharetta, GA, USA) [73]; (**D**) G-Eye endoscope (NaviAid G-EYE, SMART Medical Systems Ltd., Ra’anana, Israel) (Courtesy of PENTAX Europe GmbH); (**E**) PillCam^TM^ Colon 1 and Colon 2 [74]; (**F**) EndoRings^TM^ (EndoAid Ltd., Caesarea, Israel) [73]; (**G**) Endocuff VISION^TM^ (Olympus Corp., Tokyo, Japan) [73]; (**H**) adjunct tools for colonoscopes designed and commercialized by OVESCO Endoscopy AG (Tübingen, Germany) (Courtesy of OVESCO Endoscopy AG).

The technology used for SC consists of a long semirigid insertion tube around 13 mm in diameter, with a steerable tip, but nevertheless more rigid than the colon, which is introduced through the anus and pushed forward to inspect the colonic wall. Colonoscope looping may occur during insertion, considerably stretching the colon, thus generating pain and potential tissue damage, or even perforation (0.1–0.3% for diagnostic colonoscopies) [34,35] (Figure 2A). Furthermore, even well-experienced endoscopists are often limited by the lack of manoeuvrability, which can result in about 20% of missed polyps [36]. Due to growing incidence of CRC and of the abovementioned limitations, advanced colonoscopy techniques have been developed. The unusual shape of the colon (e.g., sigmoid stricture, stenosis, fixed sigmoid, and elongated colon), along with previous abdominal surgeries with adhesions, make colonoscopy using standard equipment extremely difficult and sometimes incomplete, diminishing its diagnostic efficiency. At least a few alternatives to the standard reusable colonoscopy technology are available in the market—as reported below—encompassing elevated diagnostic rates in comparison to SC. Of note, water-assisted colonoscopy (WAC) drew the attention of endoscopists, due to elevated patient comfort (reduced loop formation, no sedation, etc. [37,38,39,40]) and the quality of scoping (higher adenomas detection rate—ADR—and precise muscle images due to water irrigation [41]). However, limited educational background and time needed to perform WAC stand behind this procedure nowadays for its limited use in the clinical practice [42].

A single-use (sterile) endoscope developed by AMBU A/S (Copenhagen, Denmark) was presented in 2019 during the Digestive Disease Week^®^ conference in San Diego, California. The primary goal of the prototype, and in general of non-reusable endoscopes, is reducing the contamination risk [43]. However, it is likely that these devices might be not envisaged as eco-friendly, as new and more stringent polices on environmental polluters are being already announced by the EU Parliament. A regulatory clearance on the device serving as duodenoscope is pending. Moreover, AMBU A/S declared in its website that a single-use endoscope for colonoscopy and endoscopy will also be launched in 2021.

Virtual colonoscopy computed tomography (CCT, also called CT-colonography) is an alternative to SC. However, even if imaging systems are getting more and more accurate and high in resolution, the detection rate of polyps is limited and often lacking, since about 30% of the polyps are flat and obscured when using these techniques. Furthermore, sampling and characterization of tissues are not possible because they are based solely on vision and these methods are often inconclusive [44]. All in all, if the CCT visualizes a lesion, a colonoscopy still serves as diagnostic mean for further evaluation and treatment [45].

Double-Balloon Enteroscopes were initially developed for small bowel scoping, but their specific features were utilized to design Double-Balloon Colonoscopes (DBC) [46], which may be the option of choice after a failed SC. DBC are about 2 m long systems including a high-resolution endoscope and two latex balloons filled with air by using pressure pumps. Alternating push and pull movements place the gut sections on the overtube, resembling DBE mode of action [47] (Figure 2B). As elegantly demonstrated in the literature, DBC following SC resulted in the discovery of advanced neoplasia [48], colon polyps, stenosis (radiation or inflammatory) and Crohn’s disease that were not identified with the standard method [49]. Relatively shorter time of colon examination, reduced conscious sedation and the lack of fluoroscopic evaluation, in comparison with all endoscopic interventions available, stand for this technique’s effectiveness [50,51,52].

Full Spectrum Endoscopy—FUSE platform (EndoChoice Inc., Alpharetta, GA, USA) is equipped with extra optics at its end, allowing the medical specialist to view the gut with a 330 degrees angle (Figure 2C). Three cameras and LEDs snap the images and present them on three monitors. A study, comparing the effectiveness of colonoscopy instruments in adenoma detection, found that FUSE platform detected a higher number of lesions in comparison to SC (missing rate 7% vs. 41%) [53]. A very recent study found that the lesion detection rate is higher in right and middle parts of the colon [54]. Similarly, a study by Kudo et al. [55] found diminished adenoma missing rate with FUSE platform. In contrast, previous trials failed to replicate these results when compared to forward-viewing approach colonoscopy in ascending colon [55,56].

The G-Eye endoscope (NaviAid G-EYE, SMART Medical Systems Ltd., Ra’anana, Israel) has an integrated (moderately inflated) balloon serving as its bending part, which allows both the withdrawal and instrument stabilization together with flattening the haustral folds and inhibiting the slippage of the bowel (Figure 2D). A recent study by Shirin et al. [57] found that the technique yielded a higher detection rate of adenomas/polyps (ADR and PDR), including well-formed, flat and sessile serrated ones, when compared to SC. When meta-analysed with a previously published paper by Halpern et al. [58], Keulen et al. [59] discovered that ADR by means of the G-Eye endoscope is 30% higher than SC.

A possible alternative to the conventional tethered colonoscopy is represented by wireless capsule endoscopes (WCE), established in the last decade and representing an interesting non-invasive alternative to standard endoscopy [60]. WCE allows gentle inspection of the entire gastrointestinal tract without any discomfort and therefore with no need for sedation; this may encourage patients to accept gastrointestinal tract examinations thanks to its lack of invasiveness. However, WCE is a passive device moving through peristalsis and, therefore, it is not ideal for capturing images of specific areas of interest, as it cannot be stopped, oriented and navigated [61]; this limits its key-application to the small bowel. Differently, the large intestine requires adequate distension for inspection and navigation that allows visual orientation. Therefore, WCE for the large bowel (PillCam^TM^ Colon 1 and Colon 2—Medtronic Inc., Minneapolis, Minnesota, USA) inspection so far failed to show results competitive with conventional colonoscopies [62,63,64] (Figure 2E). Of note, lumen preparation is still necessary for the usage of WCE. C-Scan^®^ Cap wireless colonic capsule (Check-Cap Ltd., Isfiya, Israel) is deprived of this need, i.e., the capsule is based on X-ray technology and together with the localization data provided by means of wireless communication, it allows the creation of a 3D map of the inside colon view [65].

To increase the lesion detection rate, some adjunct tools may be placed on the top of the colonoscopes. For instance, EndoRings^TM^ (EndoAid Ltd., Caesarea, Israel) are circular add-ons stretching the gut folds when removing the colonoscopic device (Figure 2F). Such adjunctive tool was found to elevate the ADR as demonstrated in a CLEVER study [66]. A randomized trial conducted by Rex et al. [67] in 2018 revealed that the ADR was higher in case of EndoRings^TM^ usage when compared to FUSE system. Another similar adjunct tool is Endocuff VISION^TM^ (Olympus Corp., Tokyo, Japan), a single-use device using arms instead of flaps to straighten out the mucosa (Figure 2G). In a randomised trial, Endocuff VISION^TM^ was able to significantly increase the ADR if compared to SC, i.e., 35.4% vs. 20.7%, with comparable overall procedure time and without major adverse events [68]. Moreover, in the same multicentre randomized study proposed by Rex et al. [67], conducted with 1188 patients, ADR with Endocuff VISION^TM^ (adenomas per colonoscopy—APC—mean ± standard deviation: 1.82 ± 2.58), EndoRings^TM^ (1.55 ± 2.42) and standard HD colonoscopy (1.53 ± 2.33) were all higher than FUSE (1.30 ± 1.96; *p* < 0.001 for APC). In summary, forward-viewing HD instruments that dominate the FUSE system and Endocuff VISION^TM^ is a dominant strategy over EndoRings^TM^, as reported by Rex et al. [67].

Colonoscopy assisted by a transparent Cap (Reveal^®^ Distal Attachment Cap, Steris Corp., Mentor, OH, USA), attached to the tip of the endoscope, was introduced to elevate the ADR via mucosal folds flattening and minimizing a red-out, while preventing the mucosa to adhere to the lens. A meta-analysis by Nutalapati et al. [69] found that the cap improved the ADR by almost 20%, and improved the cecal intubation rate and time (CIR and CIT, i.e., rate and time of cecal intubation, defined as the passage of the colonoscope tip to a point proximal to the ileocecal valve, so that the entire cecal caput, including the medial wall of the cecum between the ileocecal valve and appendiceal orifice, is visible). However, as elegantly discussed by Frieling [70], it might be “[...] beneficial, especially for unexperienced endoscopists”, thus associated with training commitment. On the other hand, Pohl et al. [71] concluded that cap-assisted colonoscopy did not significantly improve the ADR and consequently it may be beneficial only for a percentage of endoscopists.

Similarly to novel colonoscopy-based techniques, serving for better diagnostics and therapy of CRC and elevating patient comfort during the procedure, more and more endoscopic add-on tools are being introduced in the market, such as the ones produced by OVESCO Endoscopy AG (Tübingen, Germany) [72] (Figure 2H) that provide additional therapeutic or surgical functionalities to conventional endoscopes.

Table 1 reports a summary of the distinctive features, advantages, and limitations of the aforementioned non-robotic colonoscopes and colonoscopy adjuncts, used in the clinical practice, comparing them with a quantitative analysis when possible.

## 3. Robotic Flexible Colonoscopes: Commercially-Certified Instruments

Self-propelling robotic colonoscopes are already available on the market and exhaustively described in the literature; in this section, authors also report systems that were available on the market and, therefore, robotic flexible colonoscopes that obtained commercial approval, e.g., CE mark, FDA or CFDA.

Robotic flexible colonoscopes can be classified based on the actuation principle used to negotiate the deformable and unstructured colonic tract, such as: (1) electro-mechanically actuated with a “follow-the-leader” mechanism, i.e., the NeoGuide Endoscopy System, or with an inverted-sleeve mechanism through wheels, i.e., the Invendoscope, or (2) using electro-pneumatic mechanisms, i.e., Aer-O-Scope System, ColonoSight and the Endotics System. An innovative robotic platform, worth mentioning in this section, even if applied so far only to the gastric tract, is NaviCam^®^, a robotic-assisted platform to magnetically control wireless capsule endoscopes.

**Figure 3:**
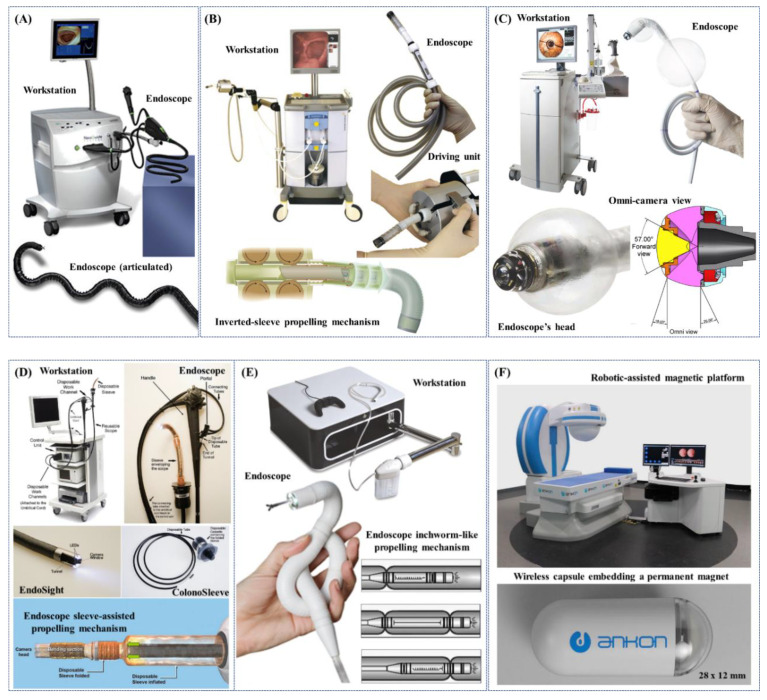
Examples of certified robotic flexible colonoscopes (commercially-available or certified but no longer on the market): (**A**) NeoGuide Endoscopy System (NeoGuide Endoscopy System Inc., Los Gatos, CA USA) [88]; (**B**) Invendoscope™ SC40 (Invendo Medical GmbH, Weinheim, Germany) [88]; (**C**) Aer-O-Scope System (GI View Ltd., Ramat Gan, Israel) [81]; (**D**) ColonoSight (Stryker GI Ltd., Haifa, Israel) [82]; (**E**) Endotics System (ERA Endoscopy Srl, Pisa, Italy) (Courtesy of ERA Endoscopy Srl) [88]; (**F**) NaviCam^®^ (Ankon Technologies Co, Ltd. Wuhan, Shanghai, China) [87].

The NeoGuide Endoscopy System (NeoGuide Endoscopy System Inc., Los Gatos, CA USA) is a FDA mark computer-assisted colonoscope consisting in a 16-segment insertion tube that controls the snake-like movement of the endoscope; each segment has two degrees of freedom (DoFs), is independent and electromechanically controlled (Figure 3A). Thanks to positions sensors at the distal tip of the endoscope and at the external base of the device, live view of the position of the scope’s tip, insertion depth and computed real-time 3D mapping of the colon, can be obtained. Computerized mapping enables the insertion tube to change the segments shape at different insertion depths in a “follow-the-leader” manner to negotiate colonic flexures in order to reduce looping and unintentional lateral forces applied to the colon wall and, thus, patient discomfort during the procedure [75,76]. NeoGuide endoscope showed successful cecal intubation with safety and effectiveness in 10 patients, with an overall procedure time, including therapeutic invention, of 34 min (range: 24–60 min), demonstrating a reduction in the looping rate thanks to the assistance of the computerized 3D mapping images [77]. Further human studies of the NeoGuide endoscope were warranted in order to improve the platform and to establish its potential for NOTES [78]. Approval of the system from the FDA was obtained in 2006 and the system was acquired by Intuitive Surgical Inc. (Sunnyvale, CA, USA) in 2009. As a result of this acquisition, some of the key-technologies were translated to Ion, a new robotic-assisted endoluminal platform developed by Intuitive Surgical Inc. for minimally invasive peripheral lung biopsy.

The Invendoscope™ SC40 (Invendo Medical GmbH, Weinheim, Germany) is a CE and FDA mark computer-assisted single-use colonoscope propelled, forward or backward, by an inverted-sleeve mechanism composed of eight drive wheels (Figure 3B). The colonoscope has a 10-mm inner sheath; a sleeve is pulled over this inner sheath, inverted at each of the respective ends (at the biopsy port and just below the endoscope deflection) and attached to a propulsion connector. The connector is then locked into an endoscope driving unit and the examination is started. A hand-held control unit is used to activate all the endoscopic and software functions. When the forward or backward buttons on the hand-held device are pressed, eight wheels in the endoscope driving unit start to move in the selected direction. The wheels grip onto the inner side of the inverted sleeve, causing the inverted sleeve and inner sheath to drive either forward or backward. The colonoscope has a unique robotically-driven tip armed with three white light emitting diode (LEDs) and a complementary metal-oxide-semiconductor (CMOS) vision chip with a field of view of 114°. The colonoscope tip can be flexed electro-hydraulically through a hand-held unit to 180° (at body temperature) in any direction and can move in circles, providing the operator with a complete view of the lumen; it also allows full retroflection for inspection of the mucosa behind colonic folds. The colonoscope has an overall diameter of 18 mm and a working length of 2000 mm (in its last version). In addition, standard functions including suction, irrigation, and insufflation are also provided along with a 3.2 mm working channel for biopsies and routine therapeutic procedures, such as polypectomy [75]. A clinical study showed a CIR of 98.4% (median time: 15 min), without any pain, in 92% of patients. Twenty-seven polypectomies were successfully performed in 23 patients [79]. However, this prototype has been replaced by a manually inserted single use device with standard flexibility and with a hand-held electrical control interface, namely the Invendoscope™ SC200 (as part of the Invendoscopy^TM^ E200 system), that obtained the CE mark certification in 2016 and in January 2018 the FDA clearance for the Invendoscopy^TM^ system E210 and for the Invendoscope^TM^ SC210. In October 2017, Invendo Medical GmbH was acquired by Ambu A/S [75].

The Aer-O-Scope System (GI View Ltd., Ramat Gan, Israel) is a CE and FDA mark pneumatically-actuated self-propelling, self-steering and disposable robotic colonoscope (Figure 3C). Active locomotion is obtained through two inflatable balloons and internal pneumatic pressure. Both balloons are inserted into the rectum and, by inflating them, the colon section in between is sealed. When CO_2_ is inflated between the two balloons, the pneumatic force pushes the frontal mobile balloon forward, minimizing the need for the operator to exert external manual pushing force, significantly facilitating its negotiation through colonic flexures. Once the mobile balloon reaches the cecum, the CO_2_ between the balloons is vented, CO_2_ is inflated between the cecum and the frontal mobile balloon, so that the pneumatic force pushes the mobile balloon backward. A 360° omni-directional high-definition vision system composed of a camera with a field of view of 57°, a dedicated optical module and LEDs are carried by the frontal mobile balloon and remotely controlled by a hand-held interface by the operator to inspect the colon; the latest Aer-O-Scope System is equipped with two working channels dedicated to treatments. To protect the intestine from possible damages, the operating pressure is monitored through electronic sensors to not exceed 60 mbar [80]. A recent study with 58 subjects shows that the Aer-O-Scope colonoscope has a CIR of 98.2% and a PDR (including all polyps larger than 5mm) of 87.5% compared to SC; in addition, no mucosal damage or adverse events were reported [81].

ColonoSight (Stryker GI Ltd., Haifa, Israel) is a self-advancing system composed of a reusable colonoscope, named EndoSight, with LEDs and a camera at the tip, covered by a wrapped disposable multilumen sheath with working channel, named ColonoSleeve, to prevent the endoscope from contact with potentially infectious agents and thus to eliminate the need for disinfection [82] (Figure 3D). The device is powered by an electro-pneumatic unit that insufflates the outer sheath to generate, by progressively unfolding it, a forward force at the distal tip enabling to pull the endoscope through the colon. This electro-pneumatic mechanism helps in reducing the overall “pushing” force required to insert the device. A multicentre trial with 178 participants showed a 90% CIR in a mean time of 11.2 ± 6.5 min. Biopsies were taken in some of the procedures and no complications, e.g., bleeding or perforation, were noted after a fortnight, thus showing a promising potential of this device over SC [82].

The Endotics System (ERA Endoscopy Srl, Pisa, Italy) is a CE mark pneumatically-driven robotic disposable colonoscope able to crawl through the colon by using two mucosal clamping devices, located at the proximal and distal ends of the probe, and a soft extension/retraction central mechanism, mimicking an inchworm-like locomotion (Figure 3E). Semiautonomous locomotion occurs by a series of consecutive steps: (1) the proximal clamp attaches to the mucosal surface, next the body of the probe elongates, (2) the distal clamp attaches to the mucosa and the proximal clamp detaches, and (3) the body contracts and the process begins again. The steerable head contains LEDs, a CMOS camera with a 140° field of view, a water and air channel for cleaning /drying the lens and for insufflation and a 3 mm working channel. The robotic device is remotely controlled by a hand-held interface through the workstation and is able to bend up to a 180° angle in every direction with very high precision, both in step-by-step mode (i.e., digital mode) and in continuous mode (i.e., analog mode), as well as electronic chromoendoscopy [75,83]. A study with 40 enrolled patients evaluated the forces applied by the Endotics System compared to the traditional colonoscope, showing that the stress pattern related to the RC was 90% lower than that of SC. All patients rated the RC as virtually painless compared to SC, ranking pain and discomfort as 0.9 and 1.1, respectively, on a scale of 0 to 10, versus 6.9 and 6.8, respectively, for the SC [84]. In a first study conducted on 71 subjects with clinical or familial risk of colonic polyps/carcinomas, the cecum was reached in 81.6% of examinations (94.3% with SC), and the average time was 45.1 ± 18.5 and 23.7 ± 7.2 min for the robotic and traditional colonoscopy, respectively. No patient required sedation during the robotic examination, compared with 19.7% of patients undergoing SC. Finally, the sensitivity and specificity of the Endotics System for detecting polyps were 93.3% and 100%, respectively, the positive predictive value 100% and the negative predictive value 97.7% [85]. In another retrospective study, senior gastroenterologists performed both traditional colonoscopy and Endotics System colonoscopy without the use of sedative agents on 276 patients. One hundred and two out of 276 Endotics RC examinations were performed in a series of patients who had undergone SC and had failed cecal intubation (difficult cases). Overall, Endotics system was successful in 93.1% of cases of incomplete SC (95% performance) [86]. Recently, a single-centre prospective pilot study was performed recruiting 56 consecutive outpatients for elective RC. Training progress in RC was assessed comparing the results of two consecutive blocks of 27 (Group A) and 28 (Group B) procedures. CIR was 92.7%, reaching 100% in Group B. Comparing the two groups, CIT significantly decreased from 55 to 22 min, whereas procedures with CIT < 20 min increased. PDR was 40% (males 62.5%, females 14.3%) and ADR was 26.7% (males 27.5%, females 14.3%). In addition, in this study, most of patients judged the procedure as mild or no distress, with high willingness to repeat the RC (92.7%) [83].

A noteworthy example of robotic-assisted endoscopic platform, even if applied only to the gastric district so far, is NaviCam^®^ (Ankon Technologies Co, Ltd. Wuhan, Shanghai, China), a robotic-assisted platform able to magnetically navigate an endoscopic wireless ingestible capsule in the stomach for gastric examinations (Figure 3F). The external static magnetic field generated by the NaviCam^®^ platform accurately controls, with 5-DoFs, a pill-size (28 × 12 mm) endoscopic capsule embedding a CMOS camera with a 140° field of view and a depth of field from 0 to 60 mm, LEDs and a permanent magnet [87]. The platform received the approval by the CFDA mark with a class III medical device registration certificate titled “Magnetically Controlled Robotic Capsule Endoscope”.

Table 2 reports a comparative analysis of the distinctive features and/or clinical outcomes of robotic flexible colonoscopes, that obtained approval and certification to be market-available.

## 4. Innovative Robotic Colonoscopes: Research Initiatives and Devices

In recent decades, several research institutes contributed to the development of novel robotic colonoscopes. Even if results look promising for opening a new way of performing painless colonoscopy, most of them are still at the research level. In the current scientific literature, there is a consistent number of review papers describing low-TRL (TRL: Technology Readiness Level) robotic colonoscopes, sometimes as a simple list of devices, sometimes classifying them based on their intrinsic features [28,89,90]. In this review paper, the authors decided to critically describe each of them as part of groups of robots with the same actuation principle, being it one of the most important robotic features towards a fully-automated robotic colonoscope, and in particular devices were classified as: (1) electric-, (2) hydraulic- or pneumatic- and (3) magnetic-actuated robotic colonoscopes.

**Figure 4:**
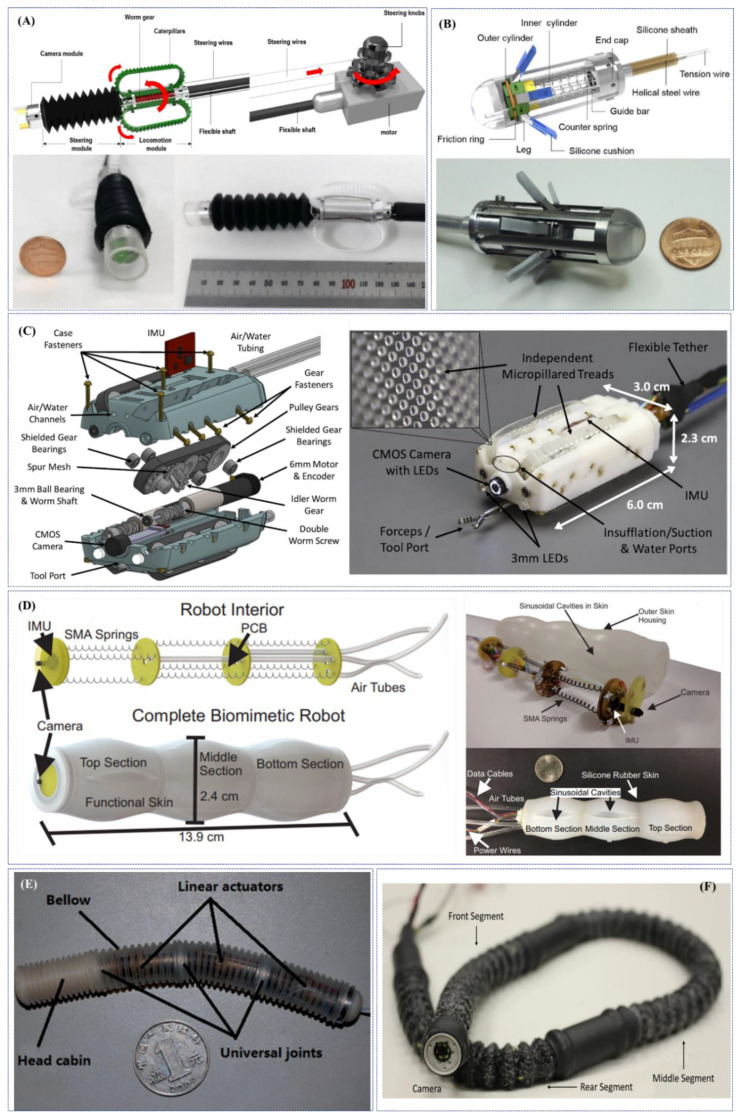
Examples of research-oriented innovative robotic colonoscopes and devices with electric actuation: (**A**) flexible caterpillar-based robotic colonoscope developed by Kim et al. [91] and Lee et al. [92]; (**B**) reel mechanism-based tethered colonoscope developed by Lee et al. [94]; (**C**) multi-DOFs sensor-enabled treaded robotic colonoscopes developed by Formosa et al. [97]; (**D**) SMA-actuated biomimetic robot developed by Ortega et al. [98]; (**E**) electrically-actuated worm-like robotic endoscope developed by Wang et al. [99]; (**F**) worm-like endoscopic robot developed by Bernth et al. (Courtesy of Prof. Hongbin Liu).

Electric actuation produces significant forces or torques through integrated mechanisms at the cost of a high-power consumption and, in the case of a wireless device, with the need of integrating a battery, a few cm^3^ in volume. However, the latter is not needed for wired robotic colonoscope in which energy, as well as other large or high-rate data and main colonoscopic services (e.g., water, air, operating channel), are provided through the tether itself. A few examples of robotic colonoscopes with electric actuation are reported in Figure 4A–F.

In 2014, Kim et al. developed a flexible caterpillar-based robotic colonoscope actuated by an external electric motor through a flexible shaft [91]. Two years later, the same research group presented an improved version of the robotic colonoscope through theoretical and experimental evaluations for the design of each component and by embedding a steering module (maximum bending angle of 178° and minimum curvature of the radius of 20 mm—experimental/simulator average error of 5.8%) (Figure 4A). Test performed in a straight excised porcine colon demonstrated reliable locomotion performance with forward and backward velocities of 5.0 ± 0.4 mm/s and 9.5 ± 0.9 mm/s, respectively (forward velocities of 6.1 ± 1.1 mm/s and 4.7 ± 0.7 mm/s in the case of 30° and 60° inclination angles, respectively). Further tests, performed in a 1 m long excised porcine colon, arranged to mimic the lower GI human anatomy, revealed a velocity of 3.0 ± 0.2 mm/s with a success rate (i.e., CIR) of 50% and a total procedure time (i.e., CIT) of 8.55 min, in case of a novice operator (#8 experiments performed). An in-vivo test performed in a live mini pig under sedation demonstrated the capability of the robotic colonoscope to arrive at the distal transverse colon, 600 mm from the anus. However, cecal intubation failed due to the mucosa structure and presence of faecal materials [92].

In 2019, Lee et al. developed a legged robotic colonoscope based on simple and reliable reel-based mechanism, actuated by an external electric motor [93] (Figure 4B). The authors demonstrated the high manoeuvrability of the colonoscopic device improved, in terms of safety, by harnessing a soft material for the six legs. In excised porcine colon the tethered robot achieved a 9.552 ± 1.940 mm/s velocity on a flat path, without any scratches or perforations on the porcine tissue [94].

Starting by a first tethered robotic capsule endoscope using micropatterned treads developed in 2012 by Sliker et al. [95], Formosa et al. presented in 2019 a novel design of a multi-DOFs sensor-enabled treaded robotic colonoscope, named Endoculus. The device presents interesting novel features such as: (1) a custom double-worm drive that removes axial gear forces while reducing radial moments, and (2) the full parameterization of gear geometries allowing size reduction via an optimization routine over design constraints [96]. Two independently-controlled motors drive micro-pillared treads above and below the device allowing for 2-DoFs skid-steering, even in a collapsed lumen. The proposed robotic colonoscope contains all the functionalities of a traditional endoscope: (1) a camera, (2) adjustable light emitting diodes (LEDs), (3) channels for insufflation and irrigation and (4) a tool port for endoscopy instruments (e.g., forceps, snares, etc.). Additionally, Endoculus carries an inertial measurement unit, magnetometer, motor encoders and motor current sensors to aid autonomous strategies in the future (Figure 4C). An in-vivo preliminary test in a live pig showed endoscopic functionalities and promising results in terms of locomotion (even if it was not able to gain consistent traction in the sigmoid area, seemingly due to excessive constriction upon the non-treaded sides of the devices). Ex-vivo tests demonstrated forward/reverse locomotion up to 40 mm/s on the colon mucosa (both not insufflated and distended), 2-DoFs steering and the ability to traverse haustral folds and functionality of endoscopic tools [97].

In the same research group, Ortega et al. in 2017 designed a soft three-modular section robot for colonoscopy with each module featured by 3-DoFs, one translation and two rotations. The robotic colonoscope uses nine independently controlled Shape Memory Alloy (SMA) springs as its actuators and a novel silicone rubber skin to provide the passive recovery force to expand the springs to their original state. In addition, it also incorporates three air tubes, one for each section, to provide forced convection reducing the cooling time of the SMA springs. In-depth FEM analysis were performed to guarantee the required mechanical behaviour (i.e., maximize traction and provide enough recovery force) and a controller unit was designed and implemented for each of the sections allowing the robot to achieve any orientation between −90° and +90° in both pitch and roll in less than 4 s with near zero steady state error. The robot uses a peristaltic motion to translate, inspired by the motion generated by the bowel, and both the peristaltic motion and the orientability of the robot were tested (Figure 4D). Tests demonstrated that the robot is able to perform a peristaltic motion with a maximum and average speed of 4 mm/s and 0.36 mm/s, respectively. Each section is also able to follow, with less than 2% overshoot and near zero steady-state error, periodic multi-input squared signals of 25° of amplitude [98].

An electrically-actuated worm-like robotic endoscope, 13 mm diameter, 105 mm in length and 22.3 g in weight, was developed by Wang et al. in 2017 [99] (Figure 4E). The lightweight robot is composed of three independent segments; each segment is composed of a linear locomotor with micromotor, turbine-worm and wire wrapping-sliding mechanism. The robot is entirely covered by an external soft bellow with excellent compatibility, designed to increase the static friction and decrease the kinetic friction in the contact state. The robot was tested in-vivo in a porcine model, demonstrating an excellent locomotion capability and safety in soft tissues, with a speed ranging between 1.62 and 2.20 mm/s and passing the entire colon with an overall time of 119 s.

Another worm-like endoscopic robot, based on an embedded electrical cable-driven actuation system, was developed by Bernth et al. in 2017 [100] (Figure 4F). The robot consists of three segments of which the two distal segments bend, thus allowing steering, while the middle segment can only extend and contract along the axial direction. By bending the two distal segments in turn, the robot can jam or wedge itself against the folds inside the colon. When one of the segments is thus jammed, the middle segment can move the rest of the robot relative to the stationary segment by either extending or contracting. Therefore, the robot itself can move forward and backward along the human colon, depending on the order in which the locomotion sequence is performed. This locomotion principle presented with this worm-like concept avoids the need for high pushing forces associated with conventional colonoscopes. Additionally, fabricated in soft material, the robot is naturally compliant and flexible, which allows the robot to pass through irregular and curved sections gently; these features can help to reduce a significant amount of pain for the patient. Based on the tests of the first prototype, this design enables the endoscope to pass through sharp bending radius, and the mechanism of the anchoring properly works well in complicated 3D and narrow colonic deformable environments.

**Figure 5:**
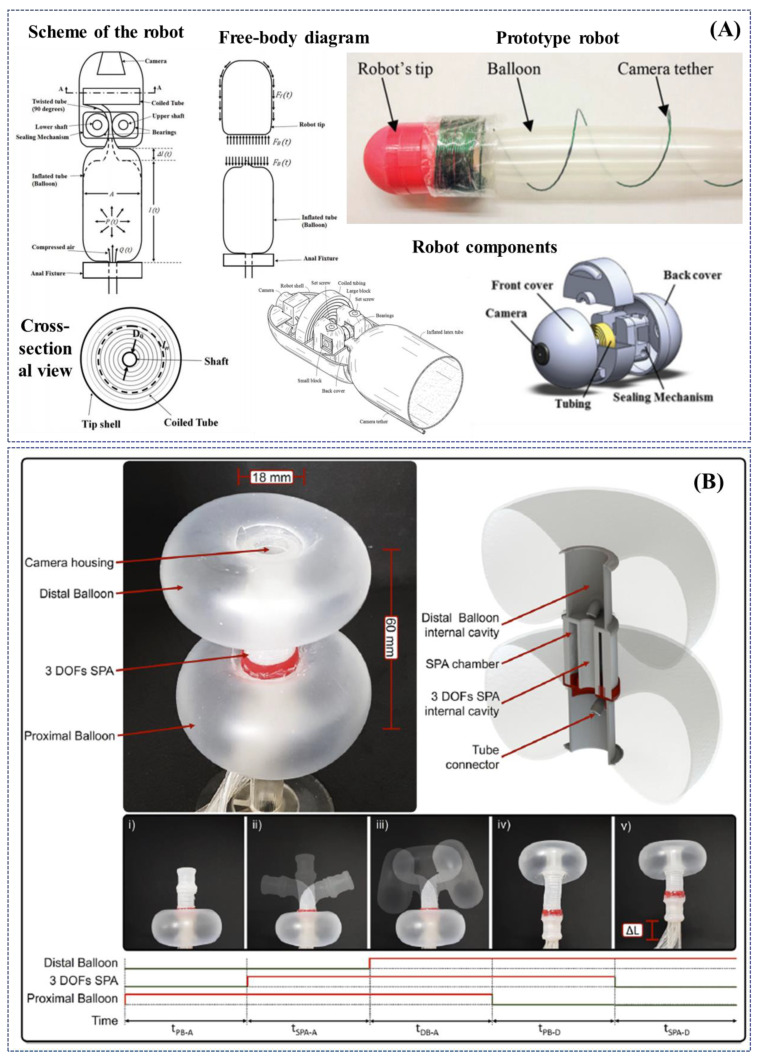
Examples of research-oriented innovative robotic colonoscopes and devices with pneumatic actuation: (**A**) self-steering pneumatically driven colonoscopy robot developed by Dehghani et al. [101]; (**B**) soft pneumatic inchworm double balloon (SPID) colonoscope developed by Manfredi et al. [102].

Another locomotion strategy, explored in the design of innovative robotic colonoscopes, based its principle on hydraulic or pneumatic actuations. These actuations are typically used in soft robotics and perfectly fit in medical applications due to a few features, such as: (1) lightweight and, usually, low inertia, (2) intrinsically safety of the soft materials, (3) reduced needs to integrate sensors and high-computational control schemes (i.e., morphological computation), (4) inert materials not affected by external disturbances, and (5) low cost, usually being disposable. Moreover, hydraulic or pneumatic actuations, at the cost of a wired connection with an external control unit and source, do not require integrating a battery for activation. A few recent examples of robotic colonoscopes with pneumatic actuation are reported in Figure 5A,B.

In 2017, Dehghani et al. developed a semiautonomous colonoscopic robot for minimally invasive procedures based on an innovative pneumatically-based locomotion approach, i.e., the tip of the robot is propelled taking advantage of a longitudinal expansion of an internal latex tube (Figure 5A). The authors performed preliminary ex-vivo experiments demonstrating that the specific robot design inherently prevents loop formation in the colon, which is recognised as the main cause of post procedural pain in patients. The robot successfully advanced for 1.5 m inside an excised porcine colon with an average speed of 28 mm/s and was capable of traversing bends up to 150 degrees. Moreover, if pressurized with 90kPa, it exerted less than 6N of normal force at the tip; a maximum force generates pressure of 44.17 mmHg at the tip, which is significantly lower than safe intraluminal human colonic pressure of 80 mmHg [101].

Another novel pneumatically actuated soft robot has been developed by Manfredi et al. in 2019. The robotic colonoscope, named SPID (i.e., Soft Pneumatic Inchworm Double balloon), consists of two inflatable distal balloons for anchorage into the colonic wall, connected by a 3-DoFs central pneumatic actuator for a bio-inspired inchworm-like locomotion and bidirectional bending. SPID, in the current version, has an external diameter of 18 mm, a total length of 60 mm and weighs 10 g. The soft and deformable structure is aimed at reducing the pressure applied to the colonic wall and consequently pain and discomfort during the procedure (Figure 5B). The colonoscopic soft robot has been tested in a deformable synthetic colon phantom, mimicking shape, and dimensions of the human anatomy. It exhibited efficient locomotion by its ability to deform and negotiate flexures and bends with an average forward speed of 2.8 mm/s (a total length of 1.4 m was covered in less than 9 min). After cecal intubation, the soft robot was withdrawn by manual traction of the tether, taking about 1 min and with an average speed of 25 mm/s; no real evaluation of the colonic mucosa was performed [102].

An interesting conceptual solution has been proposed by Consis Medical Ltd. (Beer-Sheva, Israel), an early-stage medical device company, devoted to the development of novel, semi-disposable and self-propelling robotic colonoscopes using hydraulic-aiding internal propulsion. The proposed robotic colonoscope is composed of: (1) an inverted single-use inflatable sleeve, (2) a multiple-use electronic head, embedding a working channel, a camera, light source and air and water nozzle, and (3) an external control unit. Once the electronic head is mounted and inserted into the anus, first the colon is inflated and then the device is deployed, aiding its navigation with an internal water-based hydraulic propulsion. Examination is performed withdrawing the device manually from the cecum and bending the camera with 2-DoFs [103].

**Figure 6:**
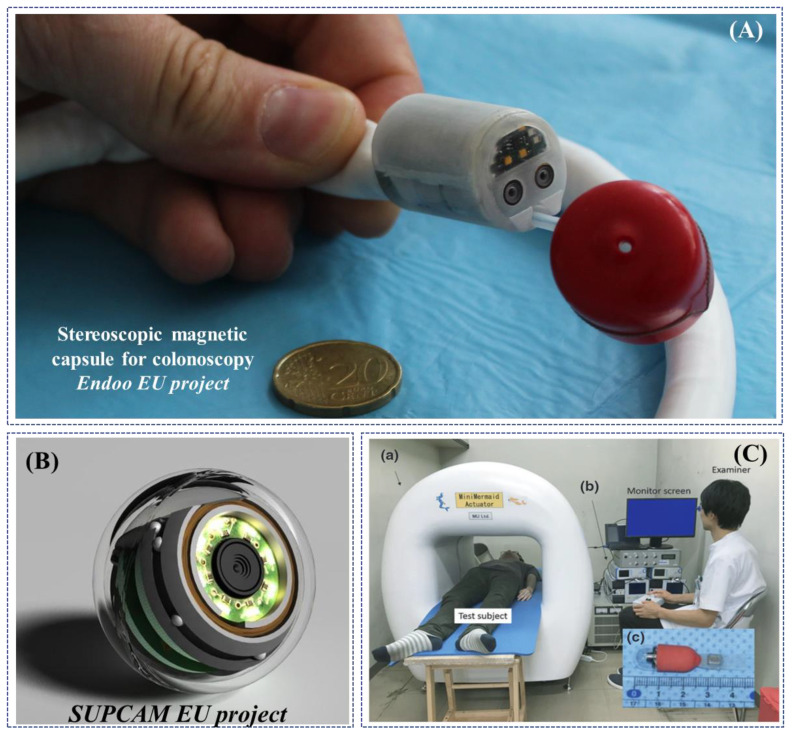
Examples of research-oriented innovative robotic colonoscopes and devices with magnetic or electromagnetic actuations: (**A**) Endoo EU project capsule (Courtesy of the Endoo consortium); (**B**) SUPCAM EU project spherical magnetic capsule for colonoscopy (Courtesy of Dr. Alessandro Tozzi); (**C**) modified PillCam^TM^ SB2 with a silicone fin for magnetic locomotion through external electromagnetic fields, developed by Nouda et al. [128].

Magnetic locomotion can be performed using either permanent or electromagnetic sources; examples of magnetic actuation and activation means applied to medical robots and applications have been presented by Sliker et al. in [104]. Magnetic actuation by permanent field sources allows for the generation of a high strength-to-size ratio magnetic field if compared to electromagnets, i.e., they can generate lager forces than electromagnets, given a comparable size and volume. The second main feature is their permanence, i.e., they can generate an electric field indefinitely, without the need for a power supply, offering an untethered magnetic field generator. The latter can be considered an advantage but also a disadvantage in an operating room since they cannot be controlled in terms of strength and they cannot be switched off. However, they can be easily customized in terms of dimensions, shapes and magnetization directions, making them suitable for different applications. Contrarily, electromagnetic field sources, if compared with permanent magnets, provide the advantage of controllability (from OFF to ON) of the generated magnetic field, increasing safety and flexibility of the system in the operating room. Nevertheless, the main disadvantages are: (1) their high size-to-strength ratio when compared to permanent magnets, (2) the need to implement control strategies, and (3) the need for a power supply to generate a magnetic field, which usually contributes to more equipment in the operating room, higher device cost and increased power demands. Finally, large electromagnetic sources present a physical limit, since the larger magnetic field that is created along the N-S pole direction can be far from the external surface of the electromagnet, and thus from the medical device if placed parallel to the magnetization direction, due to the high number of windings between the centre of the electromagnet and the external surface. A few examples of robotic colonoscopes with magnetic or electromagnetic actuations are reported in Figure 6A–C.

Magnetic-based actuation applied to endoscopic robots was first explored between 2008 and 2009 in the framework of a European FP6 project, called “Versatile Endoscopic Capsule for GI TumOr Recognition and therapy (VECTOR project)” and coordinated by Novineon Healthcare Technology Partners GmbH (Tuebingen, Germany) [105]. In the framework of this project, Ciuti et al. proposed an active locomotion approach based on permanent magnets (outside and inside the endoscopic capsule). The robotic platform for wireless capsule endoscopy combines the benefits of magnetic field strength and limited encumbrance with accurate and reliable control through the use of an external anthropomorphic robotic arm [106,107,108]. Even if the project focused on the development of magnetically-actuated wireless capsule robots, an interesting derivative outcome of the project consisted of a soft-tethered magnetically-driven capsule for colonoscopy. A proof-of-concept of the robotic colonoscope, presented by Valdastri et al. in 2012, represents a trade-off between capsule and traditional colonoscopy combining the benefits of low-invasive propulsion (through “front-wheel” locomotion) with the multi-functional tether for treatment [109]. The system has been improved in the subsequent years in terms of modelling [110,111], localization [112,113] and control [114,115,116], towards autonomous locomotion [117] and other applications [118]. A novel derivative soft-tethered magnetically-driven colonoscope was designed within a European H2020 project, called “Endoscopic versatile robotic guidance, diagnosis and therapy of magnetic-driven soft-tethered endoluminal robots (Endoo project—2015–2019)”, coordinated by the Scuola Superiore Sant’Anna (Pisa, Italy) [119]. The soft robotic colonoscope is featured by a high-definition stereo-camera with a custom-made optics, navigated by an external custom-made permanent magnet through a collaborative industrial anthropomorphic robot (COMAU SpA, Turin, Italy) [120]. A noteworthy outcome of the EU project was the development of AI algorithms to perform vision-based closed-loop control and autonomous detection and measurement of colonic lesions, e.g., polyps [121,122,123,124] (Figure 6A).

A hand-guided external electromagnetic system for a wireless colonoscope was designed in the framework of a European FP7 project, called “New cost-effective and minimally invasive endoscopic device able to investigate the colonic mucosa, ensuring a high level of navigation accuracy and enhanced diagnostic capabilities (SUPCAM project—2012–2014)”, coordinated by S.E.D. Srl (Certaldo, Italy) and supervised by Dr. Alessandro Tozzi, inventor of the omni-vision spherical capsule concept. The external electromagnetic source navigates, through a generated static magnetic field, a colonoscopic spherical-shape capsule provided with an internal permanent magnet, able to perform a 360° inspection through inner camera rotation [125,126,127] (Figure 6B).

Another significant example of robotic colonoscopic platform using electromagnetic fields, in this case alternated, has been presented by Nouda et al. in 2018. A self-propelling capsule endoscope composed by a PillCam^TM^ SB2 with a silicone fin, embedding a permanent magnet attached to it, has been tested for the first time in a healthy human volunteer. An external platform generates an alternating magnetic field that make the fin shake and thus propel the capsule, with an overall dimension of 45 mm in length and 11 mm in diameter and with a three-dimensional control. The capsule, inserted in the anus and transported with endoscopic forceps in the descending colon, was able to swim in the lumen in antegrade and retrograde directions without any damage to the mucosa [128] (Figure 6C).

Table 3 reports a comparative analysis of the distinctive technical features and/or preclinical outcomes of innovative research-based robotic colonoscopes and devices.

Keeping in mind current bottlenecks in the field of colonoscopy, mainly related to (1) pain management, (2) miniaturization, (3) smart actuation and (4) localization, interesting hints to face current technological and design challenges can originate from robotic systems developed for different medical purposes (e.g., cardiovascular interventions or drug delivery outside the gastrointestinal tract) or from bio-inspired robotic devices. The latter, as witnessed also by some commercial colonoscopes described before, appear particularly interesting for investigating locomotion strategies resulting efficient in unstructured environments. By taking inspiration from biological organisms benefitting from centuries of evolution to put in place power efficient locomotion paradigms at certain scales, it is possible to develop smart solutions for navigation across the GI tract. In this direction, Zarrouk et al. described a single actuator robot inspired by wave-like locomotion of snakes and bacteria flagella able to move forward or backward by producing a continuously advancing wave. The peculiar modular design enables locomotion over sliding terrains (as GI tract and colon could be) and against gravity by exploiting a single electromagnetic actuator. Dimensional issues (the smallest version of the robot is nearly 120 mm long and 30 mm wide) prevent at present the employment of such design inside body lumens but the principle could be inspiring for the next generation colonoscopes [129].

By taking inspiration from fungal hyphae and trailing plants, Hawkes et al. developed a growing robot characterized by extension from the tip of the body and able to change its length of hundreds of percent by also actively control the growth direction. The eversion mechanisms actuation shows some similarities with the work from Dehghani et al. [101]. Due to the intrinsically soft structure, enabled by the constitutive materials and by the pneumatic actuation, the robot design appears suitable both for fabrication in different sizes and for safe and painless interaction with surrounding tissues. The presence of a camera on the robot tip enables one to foresee the employment of such system in future endoscopic applications [130,131].

Chautems et al. recently proposed an innovative variable stiffness magnetic continuum robot eligible for different application scenarios, varying from radio-frequency cardiac ablations to interventional endoscopy in the GI tract. The device includes multiple variable stiffness modules based on a low melting point alloy, a magnetically-steerable tip and an internal working channel for an overall 2.33 mm diameter. The combination of variable stiffness polymers and magnetic tip makes it possible to accomplish complex shapes across a variety of body lumens [132].

A wide range of technologies and actuation strategies developed for smart steerable catheter, where dimension is even more relevant, can provide interesting inspiration for the development of innovative colonoscopes. Extremely interesting and innovative proposals have been made also in the field of mobile robotic systems at the meso and microscale. Such systems, despite being apparently far from the field of colonoscopy, could pave the way for a novel generation of WCE or for innovative endoscope components by making it possible to face miniaturization, powering and painless interaction issues. A wide plethora of untethered capsule robots have been proposed in recent years not only for colonoscopy but also for biopsy and drug delivery [133]. Interesting designs in this sense have been reported by Don et al. by combining magnetic actuation (both for orientation and biopsy mechanism activation), soft structure and needle-based biopsy [134]. Finally, inspiring in-vivo results were recently reported by Abramson et al. who proposed an ingestible self-orienting capsule robot for targeted, controlled release of biomacromolecules such as insulin across the GI wall [135].

## 5. Artificial Intelligence: An Enabling Factor for Enhancing Robotic Colonoscopy

Computational techniques can assist procedures in a number of ways, such as by: (1) supporting the detection and classification of disease through image analysis, (2) allowing mapping and navigation of the endoluminal environment and estimating which regions have been observed, and (3) permitting measurement of structures to support computer-aided decision making. While these application areas have been explored for several decades, it is only in recent years and through the emergence of AI systems based on data, rather than hand-crafter modelling, that the robustness of algorithms is reaching clinical translation in endoscopy. The rapid advances of AI in endoscopy in recent years have seen all major endoscopic equipment providers emerge with solutions for AI-assisted endoscopy.

**Figure 7:**
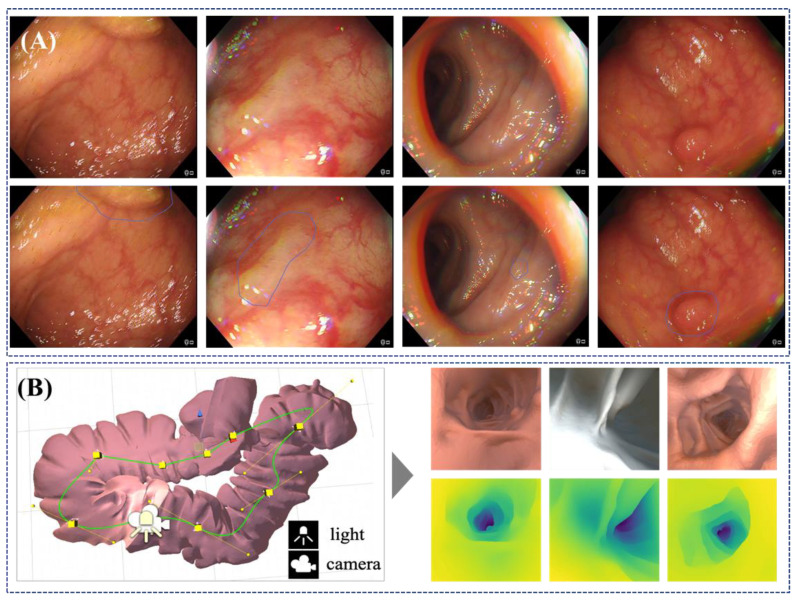
Illustration of the use of AI techniques on endoscopic images. (**A**) Shows an AI model that automatically highlights regions in the endoscopic images which are potentially occupied by a polyp; (top) original images, and (bottom) images with AI predicted regions (Courtesy of Prof. Danail Stoyanov). (**B**) Shows simulation data generated for a virtual colon, which can be used toward unsupervised AI models that can predict information that is not normally available such as depth. The image on the left shows examples of synthetic images from the simulation and depth maps predicted using an AI model where the darker colour illustrates further away from the image, whereas the image on the right shows examples of rendered RGB images with corresponding depth maps generated along camera path [172].

The most active area of AI development in endoscopy is the detection and classification of lesions, in particular colonic polyps [136,137] but also a growing number of studies with very promising results in upper GI applications like Barret’s detection [138] and squamous cell neoplasia [139]. While various endoscopic image understanding methods have been explored for a long time [140], deep-learning based techniques have shown the capability to turn algorithms into clinically valuable computer-aided diagnostic (CAD) tools [141,142,143]. There is a growing number of studies indicating that CAD systems can perform at least as well if not even better than expert endoscopists [138], though additional validation and understanding of the clinical impact is still, without doubt, needed. Such endoscopic AI systems rely on large quantities of image or video data where human observers have annotated lesions to some degree either by denoting the presence of a polyp or by delineating its position and shape within the image (Figure 7A) [124,144,145]. Labelling is a time-consuming task, and this is a current system bottleneck because experts have limited bandwidth to perform annotation, which is necessary to train the AI models. As a result, the majority of published studies in endoscopic AI systems utilise thousands of images for training, which is still significantly less than similar algorithms in vision applications, where datasets like ImageNet contain many millions of training images [146]. Some strategies around addressing this challenge are emerging in the form of open datasets, e.g., EndoVis-GIANA challenge [147] and the EAD Challenge [148] and the use of labelling farms or services, e.g., iMertit [149], as well as efforts to establish ImageNet equivalents for gastroenterology [150] or developing unsupervised learning [151].

Despite the challenges that remain around developing endoscopic AI, systems for assisting the detection of polyps are maturing into medical product lines pursued by several start-up companies, for example ai4gi [152], Odin Vision [153], Shanghai Wision AI [154], as well as the major medical device and imaging manufacturers, such as GI Genius™ Intelligent Endoscopy Module [155,156,157,158,159]. Despite being available on the market with regulatory approved products, quality assurance and appropriate UX for working with the clinical team are still issues that require resolution [160]. It is also likely that the next advances in CAD support algorithms will be in the disease identification or classification field, with some systems already emerging [161], where there is an opportunity to enhance clinical workflow and reduce costs/needs for histopathological analysis. Some preliminary studies on the opportunities for cost saving and the potential changes to recommended clinical practice with CAD are emerging [162] as are opportunities for CAD to assist the standardization of services across clinical sites and units [163].

A classic but yet unsolved problem remains in the use of AI systems to enhance endoscope navigation and the clinician’s localization within the GI tract by using image data or a combination of image and positional sensors. The importance of this capability is that it may enable quantitative measurement of the observed tissue and detection or regions that have not been observed and hence cleared as healthy. This is a longstanding area of research in all minimally invasive surgery and procedures [164]. The problem can be decomposed into a joint problem of estimating the shape of the GI tract during an endoscopic investigation and also estimating the pose of the endoscopic camera within. Classical techniques to tackle the problem [165,166] have now been superseded by supervised deep learning methods for endoscopy/laparoscopy [167,168], which estimate the 3D geometry of the anatomy. Supervision is typically achieved through simulated environments or ex-vivo scenes where ground truth can be generated using another technique, such as tomographic scanning or structured light [169]. Some preliminary results on deep learning using unsupervised strategies have also been reported by Münzer et al. [170]. While the full navigation problem is still challenging to solve robustly, some interesting approaches have emerged to support subtasks that require monocular depth estimation (Figure 7B) [171] and on measurements of polyp size which are used to make a decision on whether to perform polypectomy or not [172].

## 6. Discussion and Conclusions

Since its introduction in the clinical practice, colonoscopy has been honed to a highly effective diagnostic and therapeutic modality that has transformed provision of GI healthcare and it became one of the main pillars of an entire specialty. However, the main advantage of the procedure remains one of its main drawbacks, i.e., the need to push a flexible endoscope from the anal orifice to the caecum with all that entails in terms of discomfort and complications for patients. We are living in an era of change, both in terms of fast pace developments in precision/personalised medicine, as well as technological delivery since the dawn of the millennium. We are living the “belle epoque” of start-ups, digitalisation and resurge of AI; furthermore, there is a lot of interest in ways to eliminate human impact on the environment and reduce the mistakes in healthcare services delivery together with some added efforts to “equalise” healthcare provision across the globe. What is perhaps most relevant at the moment is the fact that novel infectious agents, e.g., SARS-CoV-2, are disrupting our normal living conditions, the global economy and they are posing a major threat to human life either directly or indirectly through an immense strain placed on the shrivelling healthcare resources, and this calls for implementation of measures such as “social and medical distancing”.

In this environment, the interface between machines/robots and humans, present at the receiving end of services, is becoming smoother and their resistance of acceptance is curbed. In this era, the use of robotic adjuncts or full robotization/automatization of the procedure are major contributions to explore, such as for guaranteeing the “social and medical distancing”. We should not forget though that there is a significant majority of workers, including healthcare professionals, that would like to see quotas to protect the human delivery of care in the face of increasing automation and the risk this could pose to jobs. Indeed, tasks no longer needed in this area could be offset by an upswing in other areas, such as more quality time spent with patients in national health systems. Nevertheless, it is envisaged that a robotic colonoscopy will allow enhanced precision and visualization enabling, therefore making possible therapeutic procedures that were otherwise considered too challenging without a robotic instrument. However, complaints of system malfunctions and reports of patient injuries may appear with widespread use and that could lead to lawsuits against stakeholders, which include the device manufacturer, the hospital or institutions and their staff, as well as the surgeons and their associates. Each of these stakeholders involved in robotic surgery is responsible to uphold the highest level of training and care available to help patients achieve the desired outcomes.

## Figures and Tables

**Table 1:** Summary of the distinctive features, advantages, and limitations of non-robotic colonoscopes and colonoscopy adjuncts, available in the clinical practice.

Device	Distinctive Features	Advantages	Limitations	Ref.s
Standard Colonoscopy (SC)	Long semirigid instrument (~13 mm in diameter and ~1400 mm in length) with a 2-DoFs cable-driven steerable tip, manually introduced through the anus and pushed forward and backward to inspect the colonic wall (+ 1-DoF axial-roll).	Current reference standard for diagnosis and treatment; diagnosis and treatment in the same session; manual fine control of the endoscope tip.	Requires unpleasant laxative preparation, sedatives, and analgesia; uncomfortable procedure due to insufflation and tissue-colonoscope interaction; highly dependent of endoscopist training and ability; looping and potential risk of perforation (0.1–0.3% for diagnostic colonoscopies).	[31,32,33,34,35,36]
Virtual Colonoscopy Computed Tomography (CCT)	Medical imaging diagnostic procedure using x-rays to compute 3D reconstructed endoluminal views of the colon.	Alternative to conventional colonoscopy to diagnose disease, e.g., polyps and diverticulosis, without discomfort generally caused by colonoscope-lumen interaction.	Requires unpleasant laxative preparation; only CT-based morphological tissue analysis; uncomfortable procedure due to insufflation; sedatives, and analgesia often required; no tissue treatment or surgery; PDR limited (30% of the polyps are flat and obscured).	[44,45]
Double-Balloon Colonoscopy (DBC)	About 2 m long system including a high-resolution endoscope and two latex balloons filled with air by using pressure pumps for easing navigation.	Relatively shorter time of colon examination, and reduced conscious sedation if compared to SC; used in the cases of technical difficulties, e.g., loop formation, long colonic segments, or suspected adhesions, resulting in the discovery of advanced neoplasia, colon polyps, stenosis and Crohn’s disease, that were not identified with SC.	Same of SC (often, with reduced discomfort, looping and risk of tissue damage); lack of fluoroscopic evaluation.	[46,47,48,49,50,51,52]
Full Spectrum Endoscopy (FUSE) (EndoChoice Inc., Alpharetta, GA, USA)	Flexible colonoscope with extra optics (i.e., three 4K Ultra HD cameras), allowing to view the gut with a panoramic 330° FoV (behind and into folds).	Maintaining standard features and functions of SC (e.g., 3.8mm working channel), FUSE demonstrated a higher lesions detection rate (mainly in the right and middle parts of the colon), compared with SC (missing rate 7% vs. 41%).	Equivalent to SC; trials failed to replicate higher performances if compared to forward-viewing approach colonoscopy in ascending colon; lower APC than SC (1.30 ± 1.96 vs. 1.53 ± 2.33).	[53,54,55,56,67]
G-Eye Endoscope (NaviAid G-EYE, SMART Medical Systems Ltd., Ra’anana, Israel)	Flexible colonoscope with an integrated inflatable balloon at its distal portion.	Balloon inflation allows, during withdrawal: (1) straightening and flattening of haustral folds, (2) inhibiting slippage of the bowel, and (3) instrument stabilization and centralized optics; higher ADR and PDR, including well-formed, flat, and sessile serrated ones, if compared to SC.	Equivalent to SC due to the same forward procedure.	[57,58,59]
Wireless Capsule Endoscopes (PillCam^TM^ Colon 2) (Medtronic Inc., Minneapolis, Minnesota, USA)	Pill-size wireless screening tools (~11 mm in diameter and ~32 mm in length) with a sub-VGA, adaptive 4 to 35 fps, 172° FoV, and ~0–30mm DoF frontal/rear CMOS double cameras with synchronized activated LEDs.	Minimally-invasive and painless; high-patient tolerability; negligible risk of perforation.	Requires unpleasant and aggressive laxatives preparation; low-accuracy and reliability for diagnosis; inability to control the capsule; inability to perform therapy and treatment.	[60,61,62,63,64,65]
EndoRings^TM^ (adjunct) (EndoAid Ltd., Caesarea, Israel)	Two layers of flexible, soft circular rings - gently flattening folds during withdrawal for a clear view.	Improved visibility, scope centring and control of the endoscope during withdrawal and tissue resection; elevate the ADR in comparison with the FUSE.	Not recommended in cases of acute, severe colitis or of known colonic strictures; performance subject to training and experience.	[66,67]
Endocuff VISION^TM^ (adjunct) (Olympus Corp., Tokyo, Japan)	Disposable add-on, using arms instead of flaps, to straighten out the mucosa.	Increased ADR if compared to SC, i.e., 35.4% vs. 20.7%, with comparable overall procedure time and without major adverse events. Higher APC than EndoRings^TM^ (1.82 ± 2.58 vs. 1.55 ± 2.42).	Not recommended in cases of acute, severe colitis or of known colonic strictures; performance subject to training and experience.	[67,68]
Transparent Cap (adjunct) (Reveal^®^ Distal Attachment Cap, Steris Corp., Mentor, Ohio, USA)	Transparent distal attachment, connected to the colonoscope’s tip and designed to elevate the ADR via mucosal folds flattening and minimizing a red-out, while preventing the mucosa to adhere to the lens.	Doubtful improvement of ADR, CIR and CIT; a study reports a higher ADR of almost 20% and improved CIR and CIT.	Performance subject to training and experience.	[69,70,71]

**Table 2:** Summary of the technical distinctive features and/or clinical outcomes (if available) of robotic flexible colonoscopes, that obtained approval and certification to be market-available (commercially-available or no longer on the market). NaviCam^®^ has not been detailed in this table because it is not applied yet in colonoscopic procedures.

Device	Actuation Principle	Technical Distinctive Features	Clinical-Oriented Features, Studies, and Clinical Outcomes	Ref.s
NeoGuide Endoscopy System (NeoGuide Endoscopy System Inc., Los Gatos, CA USA)	Electro-mechanical actuation with a “follow-the-leader” mechanism.	16-segment insertion tube that controls the snake-like movement of the endoscope; each independent and electromechanically-controlled segment has 2-DoFs; position sensors at the distal tip of the endoscope and at the external base of the device to obtain live view of the position of the scope’s tip, insertion depth and computed real-time 3D mapping of the colon.	Computerized mapping enables the insertion tube to change the segments shape at different insertion depths to reduce looping and unintentional lateral forces and, consequently, patient discomfort; successful and safe (reduction in the looping rate) cecal intubation in 10 patients, with a CIT (with therapeutic invention) of 34 min (range: 24–60 min); FDA obtained in 2006, and acquisition by Intuitive Surgical Inc. in 2009; no longer available on the market and technology translated to Ion, a robotic-assisted endoluminal platform for minimally invasive peripheral lung biopsy.	[76,77,78]
Invendoscope™ SC40 (Invendo Medical GmbH, Weinheim, Germany), then (AMBU A/S, Copenhagen, Denmark)	Electro-mechanical actuation with an inverted sleeve mechanism.	Computer-assisted single-use colonoscope propelled, forward or backward, by an inverted-sleeve mechanism composed of eight drive wheels; robotically-driven tip with LEDs and a CMOS 114° camera, electro-hydraulically flexed through a hand-held control unit to 180° in any direction with full retroflection; diameter of 18 mm and working length of 2000 mm with standard functions including: (1) suction, (2) irrigation, and (3) insufflation with a 3.2 mm working channel, also used for conventional therapeutic procedures.	CIR of 98.4% (median time: 15 min), without any pain, in 92% of patients. 27 polypectomies successfully performed in 23 patients; Invendoscope™ SC40 replaced by a manually inserted single use device with standard flexibility and a hand-held electrical control interface, namely the Invendoscope™ SC200 (as part of the Invendoscopy^TM^ E200 system); latter, obtained the CE mark in 2016 and in January 2018 the FDA clearance for the Invendoscopy^TM^ system E210 and for the Invendoscope^TM^ SC210; no longer available on the market, acquisition by Ambu A/S in 2017.	[76,80]
Aer-O-Scope System (GI View Ltd., Ramat Gan, Israel)	Electro-pneumatic actuation.	Self-propelling, self-steering and disposable robotic colonoscope with navigation obtained through two sealed inflatable balloons and internal pneumatic pressure (inflation of CO_2_) for pushing the frontal mobile balloon forward and backward; hand-held control unit to teleoperate the colonoscope’s tip with: (1) a 360° omni-directional HD vision system with a 57° FoV camera, (2) LEDs, and (3) two working channels for conventional therapeutic procedures in the latest version; monitored pressure, through electronic sensors, ≤60 mbar.	In-vivo study with 58 patients proved a CIR of 98.2% and a PDR (including all polyps larger than 5 mm) of 87.5% compared with SC, and no mucosal damage or adverse events were reported; FDA mark obtained in 2014 (and CE mark in Europe); currently available on the market.	[81]
ColonoSight (Stryker GI Ltd., Haifa, Israel)	Electro-pneumatic actuation.	Self-advancing system composed of: (1) a reusable colonoscope (EndoSight), with LEDs and a camera, covered by (2) a wrapped disposable multi-lumen sheath with working channel (ColonoSleeve), to prevent infection and eliminate the need for disinfection; powered by an electro-pneumatic unit that insufflates the outer sheath to generate, by progressively unfolding it, a forward force at the distal tip enabling to pull the colonoscope.	Electro-pneumatic mechanism helps reducing the overall “pushing” force; multicentre trial with 178 participants showed a 90% CIR in a mean time of 11.2 ± 6.5 min; biopsies taken in some of the procedures and no complications, e.g., bleeding or perforation, noted, thus showing promising potential over SC; FDA achieved in 2008, no longer available on the market.	[82]
Endotics System (ERA Endoscopy Srl, Pisa, Italy)	Electro-pneumatic actuation.	Remotely-controlled (by a hand-held control unit) disposable colonoscope able to semi-autonomously crawl the colon by using two mucosal clamping modules, located at the proximal and distal ends of the probe, and a soft extension/retraction central mechanism, mimicking an inchworm-like locomotion; steerable head, able of a 180° bending angle and, containing: (1) LEDs, (2) a CMOS camera with a 140° FoV, (3) a water and air channel for cleaning/drying the lens and for insufflation, and (4) a 3 mm working channel for conventional therapeutic procedures.	A single-centre prospective pilot study was recently performed with 56 consecutive outpatients (two consecutive blocks of 27—group A—and 28—group B—procedures); CIR was 92.7%, reaching 100% in group B; comparing the two groups, CIT significantly decreased from 55 to 22 min, whereas procedures with CIT < 20 min increased; PDR was 40% (males 62.5%, females 14.3%) and ADR was 26.7% (males 27.5%, females 14.3%); most of patients judged it as mild or no distress, with high willingness to repeat the robotic procedure (92.7%); system available on the market with CE mark obtained in 2011.	[86]

**Table 3:** Summary of the distinctive technical features and/or preclinical outcomes of innovative research-based robotic colonoscopes and devices.

Device	Actuation Principle	Technical Distinctive Features	Clinical-Oriented Features, Studies, and/or Preclinical Outcomes	Ref.s
Kim et al. 2014, Lee et al. 2016 Flexible caterpillar-based robotic colonoscope	Electric actuation.	Flexible caterpillar-based robotic colonoscope, actuated by an external electric motor through a flexible shaft, embedding a steering module (max. bending angle of 178° and min. curvature of the radius of 20 mm).	Reliable locomotion in ex-vivo straight excised porcine colon with forward and backward velocities of 5.0 ± 0.4 mm/s and 9.5 ± 0.9 mm/s, respectively (forward velocities of 6.1 ± 1.1 mm/s and 4.7 ± 0.7 mm/s in case of 30° and 60° inclination angles, respectively); ex-vivo tests, performed in a 1 m long excised porcine colon, arranged to mimic human anatomy, revealed a velocity of 3.0 ± 0.2 mm/s with a CIR of 50% and a CIT of 8.55 min, in case of a novice operator (#8 experiments performed); in-vivo tests, performed in a live mini pig, demonstrated the capability to reach the distal transverse colon, 600 mm from the anus, but in-vivo cecal intubation failed due to the mucosa structure and faecal materials.	[91,92]
Lee et al. 2018 and 2019 Reel mechanism-based tethered colonoscope	Electric actuation.	Legged robotic colonoscope based on simple and reliable reel-based mechanism, actuated by an external electric motor.	High manoeuvrability of the colonoscopic device improved, in terms of safety, by harnessing a soft material for the six legs; ex-vivo tests in excised porcine colon demonstrated a 9.552 ± 1.940 mm/s velocity on a flat path, without any scratches or perforations in the porcine tissue.	[93,94]
Formosa et al. 2019 Multi-DOFs sensor-enabled treaded robotic colonoscope	Electric actuation.	Two independently-controlled motors drive micro-pillared treads, above and below the device, for 2-DoFs skid-steering, even in a collapsed lumen; all the functionalities of a SC, i.e., (1) camera, (2) adjustable LEDs, (3) channels for insufflation and irrigation and (4) a tool port for conventional therapeutic procedures; in addition, it embeds (5) an inertial measurement unit, magnetometer, motor encoders, and motor current sensors for potential autonomous navigation.	In-vivo preliminary test in a live pig showed endoscopic functionalities and promising results in terms of locomotion (even if it was not able to gain consistent traction in the sigmoid area, seemingly due to excessive constriction upon the non-treaded sides of the devices); ex-vivo tests demonstrated forward/reverse locomotion up to 40 mm/s on the colon mucosa (both not insufflated and distended), 2-DoFs steering, and the ability to traverse haustral folds, and functionality of endoscopic tools.	[95,96,97]
Ortega et al. 2017 SMA-based three modular section soft robotic colonoscope	Electric actuation.	Each module is featured by 3-DoFs (one translation, using a peristaltic motion to translate, and two rotations); nine independently controlled SMA springs as actuators and a silicone rubber skin to passively recover force to expand the springs to the original state; three air tubes, one for each section, to provide forced convection for cooling SMA springs; orientation between −90° and +90° in both pitch and roll in less than 4 s with near zero steady state error.	In-vitro tests (rigid tube and open environment) demonstrated a peristaltic motion with a maximum and average speed of 4 mm/s and 0.36 mm/s, respectively.	[98]
Wang et al. 2017 Worm-like lightweight robotic colonoscope	Electric actuation.	Lightweight robot (13 mm diameter, 105 mm in length and 22.3 g in weight) with three independent segments, each one composed of a linear locomotor with micromotor, turbine-worm and wire wrapping-sliding mechanism; covered by an external soft bellow with excellent compatibility, designed to increase the static friction and decrease the kinetic friction in the contact state.	In-vivo tests in a porcine model, demonstrating an excellent locomotion capability and safety in soft tissues, with a speed ranging between 1.62 and 2.20 mm/s and passing the entire colon with a CIT of 119s.	[99]
Bernth et al. 2017 Cable-driven actuated worm-like robotic colonoscope	Electric actuation.	Worm-like endoscopic robot, based on an embedded electrical cable-driven actuation system; composed of three segments: the two distal segments bend, allowing steering, while the middle segment extends and contracts along the axial direction for forward and backward locomotion.	Efficient navigation through sharp bending radius curves and proper anchoring in complicated 3D and narrow colonic deformable environments; locomotion strategy avoids high pushing forces associated with SC; fabricated with soft material thus, compliant and flexible for gently passing through irregular and curved sections (potential reduced pain for patients).	[100]
Dehghani et al. 2017 Semiautonomous pneumatically-driven robotic colonoscope	Pneumatic actuation.	Propulsion taking advantage of a longitudinal expansion of an internal latex tube; lightweight and low inertia colonoscopic robot.	Preliminary ex-vivo tests, in excised porcine colon, demonstrated inherently prevention of loop formation (i.e., general cause of pain); successful advancement of 1500 mm, average speed of 28 mm/s and capability of traversing bends up to 150 degrees; if pressurized with 90kPa, it exerted less than 6N of normal force at the tip; a maximum force generates pressure of 44.17 mmHg at the tip (significantly lower than safe intraluminal human colonic pressure, i.e., 80 mmHg).	[101]
Manfredi et al. 2019 Soft pneumatic inchworm-like double balloon colonoscope (SPID)	Pneumatic actuation.	Two inflatable distal balloons for anchorage into the colonic wall, connected by a 3-DoFs central pneumatic actuator for a bio-inspired inchworm-like locomotion and bidirectional bending; external diameter of 18 mm, total length of 60 mm and weight of 10 g.	Soft and deformable structure aimed at reducing the pressure applied to the colonic wall and consequently pain and discomfort during the procedure; tested in a deformable in-vitro synthetic colonic phantom, mimicking shape and dimensions of the human anatomy; efficient navigation with an average forward speed of 2.8 mm/s (a total length of 1.4 m was covered in less than 9 min); manual withdrawal, pulling the tether with an average speed of 25 mm/s, in about 1 min.	[102]
Consis Medical Ltd. Semi-disposable and self-propelling robotic colonoscopes	Hydraulic actuation.	Semi-disposable and self-propelling robotic colonoscopes using hydraulic-aiding internal propulsion; composed of: (1) an inverted single-use inflatable sleeve, (2) a multiple-use electronic head, embedding a working channel, a camera, light source and air and water nozzle, and (3) an external control unit; once the electronic head is mounted and inserted into the anus, first the colon is inflated and then the device is deployed, aiding its navigation with an internal water-based hydraulic propulsion.	Hydraulic-aiding internal propulsion allows to gently approach colonic curves with a potentially-lower stress, and thus pain; examination performed withdrawing the device manually, pulling the tether and bending the camera with 2-DoFs.	[103]
Ciuti et al. 2010, Valdastri et al. 2012 VECTOR European project	Magnetic actuation (permanent magnets).	Magnetic-based accurate locomotion of wireless and soft-tethered capsules; use of permanent magnets embedded into the capsule and as the external source controlled by a robotic arm; continuous upgrade of the soft-tethered system in terms of modelling, localization and control towards autonomous locomotion.	Wired solution represents a trade-off between capsule and SC combining the benefits of low-invasive navigation (through “front-wheel” locomotion) with the multifunctional tether for conventional treatment; ex-vivo tests in explanted porcine colon (length of 850 mm) performed by 12 users with six to eight coloured beads, measuring 5 mm in diameter, randomly installed (number and position) along the internal surface of the colon; mean percentage of identified beads of 85 ± 11% (range 64–96%) and identified beads successfully removed; mean completion time, i.e., inspection and bead removal, of 678 ± 179 s (range 384–1082 s); preliminary in-vivo tests in pigs demonstrated an average distance travelled of 800 ± 40 mm in an average time of 900 ± 195 s, including the time devoted to inserting the tool into the dedicated channel and operating the instrument.	[105,106,107,108,109,110,111,112,113,114,115,116,117,118]
ENDOO European project Soft-tethered stereoscopic robotic capsule colonoscope	Magnetic actuation (permanent magnets).	Soft-tethered magnetically-driven colonoscope with a Full-HD 170° FoV and 3–100mm DoF stereo-camera with a custom-made optics navigated by an external permanent magnet through a collaborative industrial anthropomorphic robot; advanced AI-based tools for augmented diagnosis.	Extensive experimental sessions in ex-vivo (preclinical outcomes under publication), and tests in human cadavers.	[119,120,121,122,123,124]
SUPCAM European project Spherical-shape magnetic capsule for colonoscopy	Magnetic actuation (hybrid).	Spherical colonoscopic capsule embedding a permanent magnet and guided by an external gravity-compensated hand-guided electromagnetic system (static electromagnetic field); omni-directional view, by a single embedded camera, through 360° rotation of an internal magnetic frame into a transparent spherical shell.	Reliable navigation in ex-vivo (explanted porcine colon) and in-vitro (synthetic plastic phantom) conditions; in-vitro tests performed by five novice users, completing the task (i.e., locomotion in a ~900 mm long simple and rigid tube with curves) with a time of 44 ± 8 s (range 26–67 s).	[125,126,127]
Nouda et al. 2018 PillCam^TM^ SB2 capsule with an attached silicone magnetic fin	Magnetic actuation (hybrid).	Electromagnetic locomotion (alternating electromagnetic field through an external platform) of a 3D self-propelling capsule endoscope composed by a PillCam^TM^ SB2 with an attached silicone fin, embedding a permanent magnet; modified capsule, 45mm in length and 11mm in diameter.	In-vivo human healthy volunteer test; the capsule, inserted in the anus and transported with endoscopic forceps in the descending colon, was able to swim in the lumen in antegrade and retrograde directions without any damage to the mucosa.	[128]
